# Metabolic Syndrome and Liver Disease: Re-Appraisal of Screening, Diagnosis, and Treatment Through the Paradigm Shift from NAFLD to MASLD

**DOI:** 10.3390/jcm14082750

**Published:** 2025-04-16

**Authors:** Marin Pecani, Paola Andreozzi, Roberto Cangemi, Bernadette Corica, Marzia Miglionico, Giulio Francesco Romiti, Lucia Stefanini, Valeria Raparelli, Stefania Basili

**Affiliations:** 1 Department of Experimental Medicine, Sapienza University of Rome, 00161 Rome, Italy; 2 Department of Translational and Precision Medicine, Sapienza University of Rome, 00185 Rome, Italy; 3 Cardiology Division, Department of Biomedical, Metabolic and Neural Sciences, University of Modena and Reggio Emilia, Polyclinic of Modena, 41121 Modena, Italy

**Keywords:** metabolic dysfunction-associated steatotic liver disease, non-alcoholic fatty liver disease, metabolic syndrome, cardiovascular disease

## Abstract

Metabolic dysfunction-associated steatotic liver disease (MASLD), previously known as non-alcoholic fatty liver disease (NAFLD), encompasses a spectrum of liver diseases characterized by hepatic steatosis, the presence of at least one cardiometabolic risk factor, and no other apparent cause. Metabolic syndrome (MetS) is a cluster of clinical conditions associated with increased risk of cardiovascular disease, type 2 diabetes, and overall morbidity and mortality. This narrative review summarizes the changes in the management of people with MetS and NAFLD/MASLD from screening to therapeutic strategies that have occurred in the last decades. Specifically, we underline the clinical importance of considering the different impacts of simple steatosis and advanced fibrosis and provide an up-to-date overview on non-invasive diagnostic tests (i.e., imaging and serum biomarkers), which now offer acceptable accuracy and are globally more accessible. Early detection of MetS and MASLD is a top priority as it allows for timely interventions, primarily through lifestyle modification. The liver and cardiovascular benefits of a global and multidimensional approach are not negligible. Therefore, a holistic approach to both conditions, MetS and related chronic liver disease, should be applied to improve overall health and longevity.

## 1. Introduction

The current shift in people’s lifestyles toward unhealthy diets and sedentary habits combined with increased psychosocial stress and social inequalities, is favoring a significant increase in the burden of metabolic-related diseases. Consequently, a global silent epidemic of chronic metabolic diseases, including obesity, type 2 diabetes (T2D), non-alcoholic fatty liver disease (NAFLD), and metabolic syndrome (MetS), is ongoing worldwide. Over the past four decades, NAFLD has become the leading cause of chronic liver disease, with an estimated global prevalence of approximately 30%, and is expected to continue rising in the absence of effective prevention and treatments [1,2,3]. NAFLD encompasses a spectrum of conditions characterized by excessive fat in the liver, documented by histology or imaging, in the absence of other liver diseases or secondary causes [4,5]. MetS is a constellation of frequently coexisting metabolic abnormalities that increases the risk of cardiovascular disease (CVD), developing T2D, and overall promotes morbidity and mortality [6,7]. MetS is closely linked to insulin resistance (IR), which is common in individuals with obesity, particularly those with excess intra-abdominal or visceral adipose tissue [7,8]. One-fourth of the general population is affected by MetS, and this number is expected to continue growing. The diagnosis of MetS requires the presence of three of the following features: elevated waist circumference (WC), impaired fasting glucose (IFG) or diabetes, high blood pressure (BP), elevated levels of triglycerides (TG), and low levels of high-density lipoprotein cholesterol (HDL-C) [8,9]. Of note, NAFLD is strongly associated with the above mentioned MetS components, and it is often considered the hepatic manifestation of MetS [9,10,11]. This manuscript is a narrative review on the fundamental evidence, primarily sourced from MEDLINE, regarding the transition from NAFLD to metabolic dysfunction-associated steatotic liver disease (MASLD), with a focus on its connection to the components of MetS. Furthermore, up-to-date clinical guidelines have been incorporated to reflect the current multidisciplinary management of the clinical complexity of individuals with MASLD.

## 2. The Old and the New: From NAFLD to MASLD Definitions

After a first description of non-alcoholic steatohepatitis (1980) [12], the term “non-alcoholic fatty liver disease” (NAFLD) was coined to identify a condition of liver steatosis unrelated to alcohol in 1986 [13]. NAFLD is characterized by a progressive increase in liver fat content, without other liver disease causes or significant alcohol consumption (≥30 g/day in men, ≥20 g/day in women) [4,5]. This condition encompasses a spectrum of histopathological changes from simple steatosis (non-alcoholic fatty liver, NAFL, with >5% hepatic fat accumulation but no cell injury) to steatohepatitis (non-alcoholic steatohepatitis, NASH, defined by the presence of >5% fat accumulation, inflammation, and hepatocyte injury, with or without fibrosis). Progression of fibrosis can lead to cirrhosis with all its complications, including the development of hepatocellular carcinoma (HCC) [4,5]. Indeed, the progression from NAFL to NASH is multifactorial, and the process is not always linear, as phases of stability and/or relapse can occur along the lifespan [11,14].

NAFLD has a strong, well-established link with IR, being either a cause or a consequence of MetS [15]. The pathophysiology of NAFLD is complex. Initially, the “two hits hypothesis” was proposed: the liver lipid accumulation and IR are the first drivers that are then followed by the occurrence of inflammation, mitochondrial dysfunction, and oxidative stress, which ultimately lead to disease progression and cirrhosis. However, this hypothesis does not fully explain the complex molecular and metabolic alterations of NAFLD, and it was superseded by the “multiple hit” hypothesis. The latter considers a wider range of factors in NAFLD pathogenesis, such as IR, hormone release by adipose tissue, nutrition, gut microbiota, genetic, and epigenetic factors [16].

In 2020, a group of experts sought to revise the terminology to more accurately capture the heterogeneity of NAFLD and improve patient stratification for management. They agreed that NAFLD no longer aligns with current scientific knowledge, advocating for the adoption of ‘metabolic dysfunction-associated fatty liver disease’ (MAFLD) as a more appropriate overarching term. In contrast to the NAFLD diagnosis, which relies on exclusion criteria for alcohol consumption and other potential causes of fatty liver, the diagnosis of MAFLD is based on ‘positive criteria’. MAFLD requires evidence of liver steatosis (documented by histology, imaging techniques, or blood biomarkers/scores) and the presence of a metabolic dysfunction. Metabolic dysfunction is defined by the presence of overweight or obesity (with BMI thresholds varying by ethnicity), or T2D, or, in individuals with normal weight, at least two of the following characteristics: increased WC, elevated BP, elevated plasma TG, low plasma HDL-C, pre-diabetes, elevated homeostatic model assessment of insulin resistance, or elevated high-sensitivity C-reactive protein. Notably, the exclusion of other forms of chronic liver disease or significant alcohol consumption is not required for diagnosis, allowing for the possibility of diagnosing patients with multiple concurrent chronic liver conditions [17,18]. However, the above-mentioned definition did not reach universal consensus, opening a wide debate among multinational scientific societies on the need to improve the current nomenclature.

Later, through the application of the Delphi methodology [19], a multi-societal consensus statement was reached. First, the term fatty liver disease was replaced with the term “steatotic liver disease” to encompass different causes of steatosis. Second, NAFLD was changed to MASLD, which now requires the presence of at least one out of five cardiometabolic risk factors (i.e., abdominal obesity, dysglycemia or T2D, elevated BP, low HDL-C, hypertriglyceridemia). When applying the new MASLD criteria, around 98% of individuals identified as NAFLD in a European cohort study met the new definition [20]. Therefore, the main contribution of this change in definition was to spotlight the metabolic dysfunctions as the underling core of the continuum of the disease. Third, while the concept of steatohepatitis was retained in the new term MASH (metabolic dysfunction-associated steatohepatitis), a new category, “metabolic dysfunction and alcohol-related/associated liver disease” (MetALD), was created for individuals with MASLD consuming higher quantities of alcohol per week (female range: 140–350 g/week; male range: 210–420 g/week) [20]. The new classification and diagnostic criteria have been overall well accepted by the international community since they are non-stigmatizing and are effective toward increasing awareness and improving patient identification [20]. The different definitions of NAFLD, MAFLD, and MASLD are summarized in Table 1 [4,5,18,20].

## 3. Conditions Associated with NAFLD

### 3.1. Definitions of MetS and Its Components Intertwined with NAFLD

MetS combines complex metabolic issues and risk factors that raise the risk of CVD and T2D [7,8]. The most common risk factors include abdominal obesity (measured by WC or waist-to-hip ratio, WHR), hyperglycemia (elevated fasting blood glucose or impaired glucose tolerance, IGT), high BP (elevated systolic and/or diastolic values), hypertriglyceridemia (high TG levels), and low HDL-C [8,21,22,23]. In 1998, the World Health Organization (WHO) formulated the initial official description of MetS, which encompassed essential criteria, including IR. Since direct assessment of IR is not always available in clinical settings, several indirect measures are utilized, such as IGT, impaired fasting glucose (IFG), T2D, and reduced glucose disposal under hyperinsulinemic, euglycemic conditions. According to the WHO criteria, a diagnosis of MetS is made when a patient presents at least one measure of IR along with two additional risk factors: abdominal obesity (male WHR > 0.9, female WHR > 0.85; and/or body mass index, BMI, >30 kg/m^2^); dyslipidemia (male HDL-C < 35 mg/dL, female HDL-C < 39 mg/dL; TG ≥ 150 mg/dL); elevated BP (systolic, SBP ≥ 140 mmHg or diastolic, DBP ≥ 90 mmHg or treatment for hypertension, HTN); and microalbuminuria (urinary albumin excretion rate > 20 µg/min or albumin/creatinine ratio > 30 mg/g) [22,24]. Then, the European Group for the Study of Insulin Resistance introduced the term ‘IR syndrome’. The diagnosis of IR syndrome was based on IR, defined by a fasting plasma insulin level greater than the 75th percentile, along with at least two additional criteria. These included abdominal obesity (males WC ≥ 94 cm; females WC ≥ 80 cm), dyslipidemia (HDL-C < 39 mg/dL, or TG ≥ 150 mg/dL), IGT or IFG, and elevated BP (SBP ≥ 140 mmHg, DBP ≥ 90 mmHg, or treatment for HTN). Patients with T2D were excluded [22,25]. In 2001, the National Cholesterol Education Program (NCEP) Adult Treatment Panel III (ATP III) introduced an updated definition of MetS, in which the presence of three out of the five criteria was necessary, regardless of IR. The metabolic criteria include abdominal obesity (male WC ≥ 102 cm; female WC ≥ 88 cm), IFG, dyslipidemia (male HDL-C < 40 mg/dL; female HDL-C < 50 mg/dL; TG ≥ 150 mg/dL), and elevated BP (SBP ≥ 130 mmHg or DBP ≥ 85 mmHg) [21,22]. In 2003, the American Association of Clinical Endocrinologists reintroduced the focus on IR [22,26]. In 2005, the International Diabetes Federation (IDF) proposed a new definition of MetS, with central obesity as a mandatory marker, plus two additional factors. An American Heart Association/National Heart, Lung, and Blood Institute (AHA/NHLBI) statement maintained the ATP III criteria except for minor modifications [22,23]. Overall, at that time, the most used MetS criteria were those from the NCEP/ATP III and those of the IDF summarized in Table 2. Later, to establish a standardized and universally acknowledged definition of MetS, several organizations (the IDF Task Force on Epidemiology and Prevention, AHA/NHLBI, World Heart Federation, International Atherosclerosis Society, and International Association for the Study of Obesity) worked together and finally agreed on a uniform set of threshold values for all risk factors, except for WC. According to their joint efforts, the definition of MetS requires the presence of at least three out of five established risk factors, as detailed in Table 2 [8,21,22,23]. Recently, a collaborative position paper from various Polish scientific societies, proposed a revised definition of MetS. This definition includes elevated non-HDL-C levels (as a marker of atherogenic dyslipidemia) [27].

Among all the different definitions of MetS, a recent study showed that the 2009 IDF criteria had the highest efficacy in assessing the risk of liver abnormalities, including hepato-steatosis and fibrosis [28]. Indeed, the available evidence suggests a bidirectional relationship between MetS and NAFLD, with IR as the mutual pathophysiological factor [29]. The connection between NAFLD, MetS, and IR involves interactions of liver and other endocrine organs (i.e., pancreas, adipose tissue, and muscle), impacting the development and progression of metabolic complications [29,30]. It has been reported that the prevalence of MetS in individuals with NAFLD is approximately 43%, while the prevalence of NAFLD in those with MetS is around 73% [31,32]. While MetS increases the risk of developing NAFLD in individuals without a prior diagnosis, also NAFLD can lead to MetS features, which, in turn, can worsen NAFLD [30] in a vicious circle.

#### 3.1.1. Overweight/Obesity and Their Association with NAFLD

WHO defines being overweight and obesity as conditions resulting from the excessive accumulation of body fat, which impairs health. In adults, the most common measure used to classify these conditions is BMI (kg/m^2^): as overweight if their BMI is between 25 and 29.9 kg/m^2^, and as obese if their BMI is 30 kg/m^2^ or above. BMI serves as a valuable initial screening tool for overweight and obesity regardless of age and sex, but it does not provide the most accurate assessment of excess fat. In 2022, the WHO reported that obesity and overweight impact nearly 60% of European adults, with a higher prevalence among male individuals (63%) as compared to the female counterpart (54%) [33].

Obesity is one of the most common risk factors for NAFLD [34]. In overweight and obese individuals, the prevalence of NAFLD is estimated to be approximately 70%, while the prevalence of NASH is around 33.5%. Meanwhile, among individuals already diagnosed with NAFLD, the prevalence of obesity is approximately 51.3% [31,35]. Obesity independently raises the risk of NAFLD in a linear dose-dependent manner [36,37].

Body fat distribution exhibits sexual dimorphism, resulting in distinct patterns for men and women. On average, the gynoid-pear shape (i.e., more subcutaneous adipose tissue in the gluteo-femoral depot) is typical of premenopausal women, while men have more likely an android-apple shape (i.e., higher amounts of abdominal visceral adipose tissue, VAT). However, after menopause, the sex dimorphism attenuates, with women shifting towards android-apple shape even after considering total body fat and/or age [38,39,40,41]. VAT is an independent risk factor in the development of several diseases by influencing atherogenic dyslipidemia, IR, inflammation, and the vascular system [42]. VAT releases more free fatty acids and TG, disrupting glucose metabolism, inducing cytokine production, and contributing to liver inflammation associated with NAFLD [43]. In addition, a positive correlation between android percent fat and NAFLD, and conversely, a negative correlation with gynoid percent fat has been reported [44], explaining at least partially why NAFLD occurs more frequently in men than in woman. However, after the age of 50, both sexes tend to have a similar percentage of NAFLD. While the concept of the obesity paradox (where individuals with a moderately elevated BMI, overweight or mild obesity, may exhibit a survival benefit as compared to those with a normal BMI), remains controversial, recent evidence suggests that WHR may be a more precise predictor of mortality in NAFLD patients, further underscoring the importance of adiposity distribution in determining health outcomes in this clinical scenario [45,46]. These findings underline the critical role of body weight, body fat, and its distribution in shaping NAFLD outcomes, highlighting the importance of addressing obesity in its management.

#### 3.1.2. Diabetes and Its Association with NAFLD

Diabetes mellitus encompasses a range of metabolic abnormalities, where glucose utilization is impaired and its production is excessive due to dysregulated gluconeogenesis and glycogenolysis, leading to hyperglycemia. According to the IDF, diabetes affects approximately 9.3% of the global population, and it is a matter of concern that nearly half of them are unaware of their condition. T2D is the most common form of diabetes, accounting for 90–95% of cases. It arises from a non-autoimmune, progressive decline in adequate β-cell insulin secretion, typically occurring in the context of IR and MetS [47,48].

Given the crucial role of IR in the pathogenesis of both T2D and NAFLD, it is not surprising that patients with T2D have a two-fold higher likelihood of having NAFLD than the general population. A systematic review and meta-analysis estimated the global prevalence of NAFLD in individuals with T2D at 65%, while the prevalence of NASH with significant biopsy-confirmed fibrosis was around 40% [49]. NAFLD is associated with a significant two-fold increased risk of developing diabetes, proportionally to the severity of NAFLD [50]. The increased risk of developing T2D in NAFLD may result from hepatic lipid accumulation and inflammation, driven by several contributors, such as changes in gut microbiota and permeability, adipose tissue dysfunction with altered ceramide synthesis, and heightened hepatic glucose production [51]. A recent study revealed that the transition from NAFLD to prediabetes may represent the key pathway leading to the onset of T2D [52]. On the other side, T2D represents one of the most potent risk factors for the accelerated progression of NAFLD to NASH, and cirrhosis [51]. As liver disease severity advances, IR also increases, posing additional challenges in T2D management [53]. Therefore, it is crucial to prioritize routine screening and early detection of NASH and advanced fibrosis in patients with T2D or prediabetes. Simultaneously, individuals diagnosed with NAFLD should be screened for prediabetes and T2D.

#### 3.1.3. HTN and Its Association with NAFLD

HTN is defined as office BP ≥ 140/90 mmHg, with confirmation recommended through out-of-office measurements or a repeat office measurement. A new category, “elevated BP”, defined by SBP 120–139 mmHg or DBP 70–89 mmHg, has been introduced and pharmacologic treatment is recommended in the presence of elevated global CVD risk [54]. Globally, more than 1 billion adults, between 30 and 79 years old, have HTN, but around 46% of affected individuals are unaware of their condition [55]. Clinical research has revealed a greater association between NAFLD and HTN, beyond other MetS components. NAFLD is closely associated with the onset of HTN, and elevated BP is not only linked with NAFLD but also contributes to its progression, potentially leading to liver fibrosis [56,57,58,59]. The risk of HTN in NAFLD is influenced by IR and systemic inflammation, that might contribute to the activation of the sympathetic nervous system and the renin-angiotensin-aldosterone system, both implicated in BP regulation [60,61]. HTN co-occurs in approximately 46% of NAFLD cases, and NAFLD affects approximately 49% of patients with HTN [62,63]. A recent meta-analysis revealed that hypertensive NAFLD individuals are at a significant higher risk of all-cause death and CVD mortality; risks that are even higher if HTN is not treated [63]. Therefore, efforts focused on early detection, comprehensive management, and regular monitoring are crucial for improving health and liver outcomes in patients with HTN.

#### 3.1.4. Dyslipidemia and Its Association with NAFLD

Dyslipidemia is a condition characterized by abnormal lipid levels in the blood, which may include elevated levels of total cholesterol (TC), low-density lipoprotein cholesterol (LDL-C), and TG, or decreased levels of HDL-C. Approximately 39% of adults exhibited elevated plasma TC levels worldwide [64,65]. A meta-analysis of 86 studies showed high prevalence of combined dyslipidemia among patients with NAFLD (69%) and with NASH (72%) [31]. Atherogenic dyslipidemia (i.e., elevated TG, low HDL-C, and high LDL-C) is the predominant lipid disorder among individuals with NAFLD [66]. The accumulation of liver fat in NAFLD comes from an imbalance of several pathways, including deficient uptake of circulating lipids, increased hepatic de novo lipogenesis, limited fatty acid oxidation, and altered lipid export within very-low-density lipoprotein cholesterol [67]. IR profoundly affects lipoprotein patterns in NAFLD, resulting in increased oxidative stress, inflammation, endothelial dysfunction, and ectopic lipid accumulation. The cumulative effect of these interconnected abnormalities ultimately leads to the development and progression of atherosclerotic CVD [68]. While elevated lipoprotein(a) [Lp(a)] levels are now known as a cardiometabolic marker of increased atherosclerotic CVD (ASCVD) risk [69], lower Lp(a) levels have been associated with a higher risk of steatohepatitis, advanced fibrosis, and cirrhosis in patients with NAFLD [70].

In summary, dyslipidemia can predispose individuals to develop NAFLD by boosting fat delivery to the liver and promoting IR. NAFLD alters lipid metabolism and promotes inflammation, worsening dyslipidemia. To avoid the vicious circle between lipid and liver damage, it is essential to give priority to early detection and implement comprehensive management strategies of lipid disorders.

### 3.2. Cardiovascular Disease and Its Association with NAFLD

Emerging evidence suggests that NAFLD not only indirectly (i.e., through the higher prevalence of co-existing CV risk factors) but also directly contributes to enhance CVD risk. Thus, NAFLD independently increases the risk of established ASCVD according to liver disease severity, especially at higher fibrosis stages [71]. Furthermore, a meta-analysis demonstrated a robust independent association between NAFLD and subclinical atherosclerosis, defined as elevated carotid artery intima-media thickness (cIMT)/plaques, greater arterial stiffness, presence of coronary artery calcification (CAC), and endothelial dysfunction [72]. In another meta-analysis, NAFLD was found to be linked to the development and progression of CAC [73]. Evidence from another meta-analysis reported a significant association between liver fibrosis and subclinical atherosclerosis, more pronounced with a severe degree of fibrosis [74].

Regardless of established cardiovascular risk factors and NAFLD progression, the presence of ischemic heart disease, mainly myocardial infarction, is associated with NAFLD [75]. A meta-analysis of 11 million individuals with NAFLD reported a 1.5-fold increase in heart failure in long-term follow up, independent of traditional cardiovascular risk factors [76]. Overall, individuals with NAFLD face a 45% greater risk of experiencing both fatal and non-fatal CVD. Notably, the CVD-related mortality is the leading cause of death in NAFLD, closely followed by extrahepatic malignancies and liver-related complications. Additionally, NAFLD patients are more susceptible to conditions like aortic stenosis, carotid and coronary atherosclerosis, stroke, and atrial fibrillation [77,78,79,80]. The exact underlying mechanisms behind these associations are not fully elucidated. Dysregulated hepatic lipid metabolism, systemic/hepatic IR, oxidative stress, systemic low-grade inflammation, abnormal distribution of adipose tissue (e.g., pancreas, skeletal muscle, and epicardium), atherogenic dyslipidemia, and impaired endothelial function could serve as potential risk factors that establish a connection between NAFLD and CVD [81,82,83].

**Figure 1:**
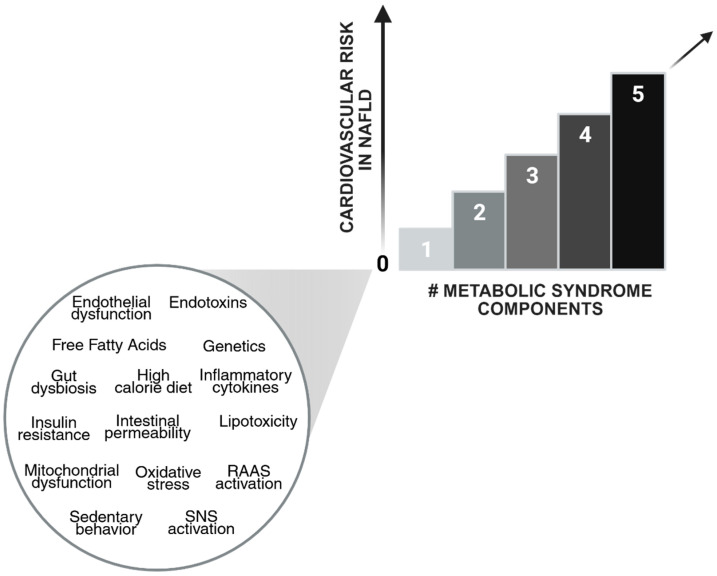
Relationship between non-alcoholic fatty liver disease (NAFLD) cardiovascular risk and metabolic syndrome (MetS) components. The components of MetS include abdominal obesity, impaired fasting glucose or diabetes, high blood pressure, elevated triglyceride levels, and low levels of high-density lipoprotein cholesterol. An increasing number of MetS components is associated with a higher risk of cardiovascular disease in patients with NAFLD. This relationship is likely mediated by various pathophysiological mechanisms, which are listed in alphabetical order from top to bottom. Created in BioRender. Stefanini, L. (2025) https://BioRender.com/aao4utq (accessed on 7 April 2025). Figure Abbreviations: RAAS, renin angiotensin aldosterone system; SNS, sympathetic nervous system.

Patients with both NAFLD and MetS are at even higher mortality and CVD risk than those without MetS. The mortality risk proportionally increases as the number of MetS components rises [84]. Therefore, to prevent the onset of CVD, it is essential to prioritize regular monitoring, which plays a central role in improving patient outcomes. The relationship between NAFLD, CVD risk, and the components of MetS is presented in Figure 1.

### 3.3. Other Clinical Conditions and Their Association with NAFLD

Several other clinical conditions have been associated with NAFLD, including obstructive sleep apnea (OSA), endocrinological abnormalities (i.e., polycystic ovary syndrome (PCOS), hypothyroidism, hypogonadism, growth hormone (GH) deficiency), sarcopenia, kidney diseases and extrahepatic cancer. OSA frequently coexists with NAFLD, and it is a documented independent risk factor for the onset and advancement of NAFLD. Intermittent hypoxia of OSA has been associated with mitochondrial dysfunction, impaired glucose and lipid metabolism, and exacerbated IR [85,86]. A recent systematic review found that after bariatric metabolic surgery, 100% of patients with mild-to-moderate OSA were free of fatty liver disease, while those with severe OSA experienced an 89% reduction in fatty liver prevalence [87]. POCS is strongly linked with an increased risk of NAFLD. In addition to elevated BMI and dysglycemia, excessive androgen levels significantly contribute to the development of NAFLD [88,89]. NAFLD is independently linked to hypothyroidism, regardless of age, sex, BMI, and other metabolic risk factors. Specifically, hypothyroidism is strongly associated with dyslipidemia and reduced hepatic β-oxidation, leading to the overproduction of triglycerides and lipotoxins. Liver-specific thyroid hormone receptor β agonists can effectively treat NAFLD, likely by enhancing lipid homeostasis and mitochondrial respiration, which may help mitigate the progression of the disease [90,91]. A recent meta-analysis confirmed the association between primary hypothyroidism and both the higher prevalence and greater histological severity of NAFLD [92]. The liver plays a vital role in the metabolism of sex steroids and the production of sex hormone-binding globulin, that regulates sex hormones activity. Thus, liver diseases, including NAFLD, are frequently associated with reproductive dysfunction. In fact, individuals with hypogonadism (i.e., a condition characterized by low levels of sex hormones) exhibit a higher prevalence of NAFLD [93]. GH and its primary mediator, insulin-like growth factor-1, are essential regulators of glucose and lipid homeostasis. They influence growth, body composition, and a wide range of physiological metabolic processes. Adult GH deficiency is often associated with MetS components and NAFLD [94,95]. In individuals with nonfunctioning pituitary adenomas, GH deficiency was associated with a two-fold higher prevalence of NAFLD, compared to those without GH deficiency [96]. Sarcopenia, a progressive loss of skeletal muscle mass and strength, is associated with a higher likelihood of NAFLD development and progression [97]. NAFLD is also an independent risk factor for developing chronic kidney disease, renal failure and CVD, and this risk increases in patients with NASH and advanced fibrosis [98,99,100]. Patients with NAFLD experience more frequently the occurrence of extrahepatic malignancies [101]. In a meta-analysis of observational cohort studies, NAFLD was associated with an elevated risk of gastrointestinal cancers (e.g., esophagus, stomach, pancreas, and colorectal), as well as other solid tumors (i.e., affecting the lung, breast, gynecological system, and urinary tract) [102]. Another meta-analysis showed that the higher prevalence of extrahepatic cancers is independent of the liver fibrosis stages. Overall, the most reported extrahepatic cancers in NAFLD were uterine, breast, prostate, colorectal, and lung cancers [103]. This risk may be increased by the association between NAFLD and MetS, which is linked to an increased likelihood of developing various common cancers [104,105,106,107]. Finally, a meta-analysis of eight observational studies (n = 56,745 individuals with NAFLD, 11% lean) demonstrated that lean NAFLD is associated with an elevated risk of hepatic, and colorectal cancers compared to non-lean NAFLD [108]. Although HCC is often linked to NAFLD-related cirrhosis, it can also appear in non-cirrhotic stages of the disease and is independently associated with other risk factors [103,109,110,111]. T2D is the most significant risk factor for HCC development in NAFLD, with the risk amplified by additional features such as obesity, HTN, and dyslipidemia [112,113]. Consequently, encouraging screening approaches for other common conditions, such as the above-mentioned, in NAFLD patients, could improve overall patient care.

## 4. Diagnosis and Screening of MASLD

As detailed in Table 1, the diagnosis of MASLD requires the identification of hepatic steatosis, the exclusion of significant alcohol consumption, and the presence of at least one of the following five cardiometabolic risk factors. If other causes of liver disease are identified, this is in line with a potential combined etiology [20]. Reassessment of existing cohort studies documented that findings from NAFLD research are directly applicable to individuals with MASLD [114,115,116,117]. Therefore, NAFL is now referred to as MASL (metabolic dysfunction-associated steatotic liver) characterized by more than 5% hepatic fat accumulation but no cell injury), and NASH has been reclassified as MASH. Additionally, cirrhosis previously linked to NASH is now considered equivalent to MASH-related cirrhosis [118,119].

Clinically, most patients with MASLD are asymptomatic. Occasionally patients experience symptoms such as fatigue, weakness, and pain in the upper right quadrant. Therefore, MASLD diagnosis often occurs incidentally during non-invasive laboratory or imaging tests (e.g., hepatic steatosis and/or altered liver enzymes). Abdominal ultrasound (US) is commonly used to document hepatic steatosis for its accessibility and cost-effectiveness [4,5,120,121]. However, the latest practice guidance from the American Association for the Study of Liver Diseases (AASLD) suggests that the controlled attenuation parameter technique may serve as a useful point-of-care method for detecting steatosis, as abdominal US remains suboptimal for its limited sensitivity across the MASLD spectrum [122].

A better assessment of hepatic steatosis can be obtained by magnetic resonance imaging-estimated proton density fat fraction (MRI-PDFF) and/or ^1^H MR spectroscopy (MRS). These imaging techniques provide information that are well correlated with pathological findings at percutaneous liver biopsy which remains the diagnostic gold standard for MASLD. As liver biopsy holds risks and costs, especially for asymptomatic patients, several alternative non-invasive methods have been proposed and are now preferred, including liver ultrasound, vibration-controlled transient elastography (VCTE), shear wave elastography (SWE), computed tomography (CT), and magnetic resonance elastography (MRE). Liver biopsies should be considered for MASLD patients at risk of steatohepatitis or advanced fibrosis, as suggested by clinical scores like the fibrosis index based on four factors (FIB-4), NAFLD fibrosis score (NFS), or liver stiffness assessed through VCTE/MRE. Additionally, liver biopsies are useful when there is a need to differentiate from other causes of hepatic steatosis or to clarify the presence and severity of concurrent liver diseases [4,5,119,122,123].

**Figure 2:**
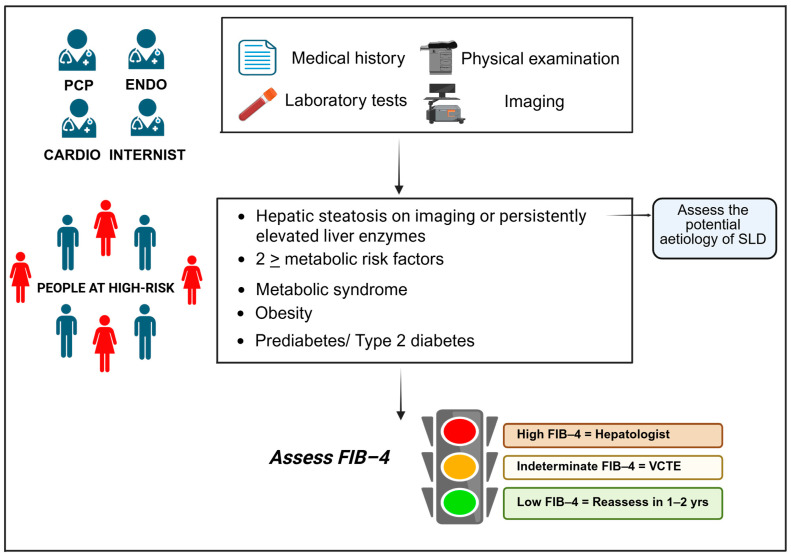
Screening approach for clinically significant liver fibrosis. Abbreviations: PCP, primary care physician; ENDO, endocrinologist; CARDIO, cardiologist; SLD, steatotic liver disease; FIB-4, fibrosis index based on 4 factors; VCTE, vibration-controlled transient elastography. Created in BioRender. Stefanini, L. (2025) https://BioRender.com/37ws4rz (accessed on 7 April 2025).

From a clinical point of view, MASL typically maintains a benign course, even though approximately 20% of patients transition towards MASH, which can lead to cirrhosis, liver failure, and even HCC [124,125]. Interestingly, the transition towards MASH is favored by the presence of MetS components, with a greater impact of T2D and obesity, than HTN and hyperlipidemia [126]. Given the well-established link between fibrosis stages and the increased risk of liver-related complications and death [127,128,129], routine screening for advanced fibrosis is recommended in high-risk populations (i.e., prediabetes or T2D, obesity, individuals with two or more cardiometabolic risk factors, hepatic steatosis detected on imaging, persistently elevated liver enzyme levels, or MetS). Non-invasive tests (NITs) commonly used in clinical practice for screening include the FIB-4, NFS, VCTE, and the enhanced liver fibrosis (ELF) score [4,5,119,122,130,131,132]. Among NITs, experts widely agree that FIB-4 stands out as the most efficient and economical approach for the initial screening of individuals with prediabetes, cardiometabolic risk factors, or T2D in both primary care and other clinical settings. FIB-4 (derived from age, alanine aminotransferase (ALT), aspartate aminotransferase (AST), and platelets) categorizes patients into low (<1.3), indeterminate (1.3–2.67), and high-risk (>2.67) fibrosis groups. As reported in Figure 2, low-risk patients should be re-evaluated in 1–2 years unless clinical conditions change. For high-risk patients, it is recommended to consult a hepatologist. For patients with indeterminate risk, where the ability of FIB-4 to accurately detect fibrosis is limited, it is advisable to consider additional tests, such as VCTE using FibroScan, ELF, SWE, MRE, or MRI-corrected T1 (cT1), if available. In the absence of these tests, referral to a hepatologist is recommended for further evaluation [119,122,123,132,133,134]. In primary care, VCTE or US-based methods (if available) are preferred over MRE for cost-effective secondary assessment. Based on an extensive multicenter study [135] and a meta-analysis [136], indeterminate-risk patients undergoing VCTE are classified into the low-risk category for advanced fibrosis if their liver stiffness measurement (LSM) falls below 8 kPa. Conversely, patients with higher LSM values should be promptly referred to a hepatologist for further evaluation and management.

The American Diabetes Association recommended screening of adults with T2D or prediabetes, particularly those with obesity, for liver issues using FIB-4, irrespective of normal liver enzyme levels. Uncertain or elevated FIB-4 outcomes should prompt liver stiffness measurement through VCTE or ELF assessment. However, referral to a hepatologist is recommended when uncertainties persist [137,138]. Indeed, FIB-4 shows diminished accuracy in young adults (<35 years old), while for older people (aged ≥65 years), higher low-risk category FIB-4 cutoffs should be considered [139,140]. As genetic factors have a significant impact on the development and progression of MASLD, screening for advanced fibrosis may be advisable for first-degree relatives of MASLD-cirrhosis patients [141,142]. Patients with MASH cirrhosis are at higher risk for liver-related complications, and they should undergo regular surveillance for HCC and esophageal varices [122,143].

Other scoring systems, incorporating NITs, have emerged to improve the identification of patients with significant fibrosis (i.e., fibrosis stage ≥ 2) [136,144,145,146]. Examples include MEFIB (comprising MRE and FIB-4), MAST (involving MRI and AST), and FAST (combining FibroScan and AST). Among them, MEFIB was proved to be more effective than MAST and FAST in detecting significant fibrosis and identifying individuals with “at-risk” MASH [147]. More recently, the diagnostic performance of FAST score was assessed, revealing notable (89%) specificity and sensitivity. These findings suggest that FAST is a cost-effective approach for identifying patients requiring further evaluation through liver biopsy or consideration for drug therapy [148]. Furthermore, machine learning models integrating clinical and metabolomic variables (such as aminotransferases, MetS components, BMI, and 3-ureidopropionate) have also been tested for improving the diagnostic accuracy of liver fibrosis [149]. Table 3 summarizes commonly used NITs and formulas for assessing liver fibrosis in MASLD, highlighting their advantages and disadvantages [150,151,152,153,154,155,156,157,158,159,160,161,162,163,164,165,166,167].

The aim of MASLD screening is to identify individuals at risk of adverse outcomes, preventing progression to cirrhosis, HCC, and ultimately reducing mortality risk. Achieving effective and sustainable long-term management of MASLD requires a holistic, multidisciplinary strategy addressing all components of MetS to customize treatment accordingly. The screening approach for clinically significant liver fibrosis is schematically presented in Figure 2.

## 5. Management of MASLD

### 5.1. Primary Prevention

The primary prevention of MASLD relies on addressing key modifiable risk factors. Lifestyle modification is the first step. Adopting a balanced, low-calorie diet rich in minimally processed foods, such as whole foods and fiber, while being low in saturated fats and refined sugars, is advisable. In fact, the Mediterranean diet has beneficial effects in preventing MASLD. In parallel, achieving and maintaining a healthy body weight, along with regular physical activity (both aerobic and resistance exercise), enhances insulin sensitivity, supports weight management, and reduces liver fat accumulation. Additionally, effective management of comorbidities (e.g., obesity, IR, IFG, T2D, dyslipidemia, HTN, gut dysbiosis, etc.) is essential for MASLD prevention. Furthermore, avoidance of smoking or hepatotoxins, such as excessive alcohol consumption and certain medications or environmental toxins, is also crucial [5,119,122,132,168]. Lastly, routine screening for MASLD in high-risk populations—such as individuals with obesity, T2D, or MetS—should be prioritized to facilitate early detection and timely intervention.

### 5.2. Treatment of MASLD

A truly effective treatment should address not just steatosis and liver injury but also the interconnected metabolic and CVD risks linked to MASLD. Consequently, lifestyle modifications are the principal and fundamental treatment approach for all patients. The clinical practice guidelines unanimously emphasize the critical significance of establishing precise weight loss targets for individuals diagnosed with MASLD who fall within the overweight or obese categories. These guidelines recommend a 7–10% weight loss for overall MASLD management but suggest exceeding 10% for significant fibrosis improvement [5,119,168]. Even a slight weight loss (3–5%) can reduce steatosis, MASH, and fibrosis [4,5]. Indeed, tailored, well-structured weight loss and exercise programs offer greater advantages in managing MASLD when compared with conventional counseling approaches [169,170].

The adoption of the Mediterranean dietary pattern is a recommended approach for treating MASLD due to its effectiveness in reducing hepatic steatosis and inflammation while also providing a huge range of health benefits beyond weight loss [5,132,168,171]. Conversely, ultra-processed foods and sugary beverages, as significant sources of saturated fat, refined carbohydrates, and fructose, exacerbate the risk of MASLD, along with the heightened risks of MetS, MASH, and significant fibrosis in individuals with MASLD [172,173,174]. Additionally, observational studies validate that the consumption of red and processed meat increases MASLD risk [175], while dietary polyphenols provide protective effects [176,177]. However, the AASLD strongly advises individuals with clinically significant hepatic fibrosis to completely abstain from alcohol consumption [122,178]. Furthermore, patients with MASLD should be strongly advised to completely avoid tobacco smoking. In fact, smoking not only elevates the risk of developing MASLD but also raises mortality rates in MASLD patients [179,180,181]. Sedentary behavior independently predicts MASLD development, while exercise is a well-established method for reducing liver fat accumulation [182]. The effectiveness of both aerobic and resistance training in improving MASLD is noticeable when engaging for approximately 40 min per session, three times a week [183]. However, it is important to acknowledge that the optimal duration of physical activity (ideally >150 min per week of moderate-intensity or >75 min per week of vigorous-intensity physical activity) should be tailored to the individual [119].

Beyond lifestyle modifications, pharmacological options to address MASLD are currently under investigation. Resmetirom, a thyroid hormone receptor-β agonist, is the first drug approved by the Food and Drug Administration for treating non-cirrhotic MASH with moderate-to-advanced hepatic fibrosis, as add-on lifestyle modifications. The recommended dosage of resmetirom is 80 mg daily for patients weighing less than 100 kg and 100 mg daily for those weighing 100 kg or more. It can be taken with or without food. Resmetirom should not be used in patients with strong CYP2C8 inhibitors (such as gemfibrozil) or OATP inhibitors (such as cyclosporine). When co-administered with moderate CYP2C8 inhibitors (e.g., clopidogrel), the dose requires adjustments (60 mg or 80 mg, depending on the patient’s body weight). Patients taking resmetirom should be regularly monitored for hepatotoxicity and gallbladder-related adverse events. Additionally, concomitant use with certain statins may require dose adjustments of the statin and close monitoring for potential statin-related side-effects [184,185].

While resmetirom is currently approved only in the United States, MASH, the progressive subtype of MASLD, remains without a specific treatment in Europe.

However, some drugs approved for other conditions have demonstrated potential benefits for MASH in clinical trials and may be worth considering for use in relevant situations. Glucagon-like peptide-1 receptor agonists (GLP-1RAs), such as semaglutide, approved for treating T2D and obesity, hold potential for MASH patients, offering cardiovascular benefits and MASH improvement [186]. Tirzepatide, an agonist of glucose-dependent insulinotropic polypeptide and GLP-1 approved for treatment of T2D, showed promising improvements in MASH during the phase 2 randomized trial [187]. While survodutide, an investigational long-acting dual agonist of glucagon and GLP-1 receptors, also exhibited potential for enhancing MASH in a similar trial [188]. Retatrutide, a novel triple agonist targeting glucose-dependent insulinotropic polypeptide (GIP), GLP-1, and glucagon receptors, achieved significant liver fat reduction (<5%) in up to 86% of participants at the maximal dose in a randomized phase 2a trial [189]. Efruxifermin, a bivalent Fc-fibroblast growth factor 21 (FGF21) analogue, and pegozafermin, a long-acting glycopegylated FGF21 analogue, have shown significant fibrosis and MASH improvements in phase 2b randomized controlled trials [190,191]. Lanifibranor, a pan-PPAR (peroxisome proliferator-activated receptor) agonist, also demonstrated improvements in fibrosis and MASH in a phase 2b randomized controlled trial [192]. Pioglitazone, effective in enhancing MASH, is worth considering for individuals with both MASH and T2D [193]. Sodium-glucose cotransporter (SGLT-2) inhibitors show benefits in metabolic abnormalities and promise in MASLD, but their impact on liver histopathology remains incompletely elucidated [194,195,196,197]. Additionally, vitamin E may be an option for specific non-diabetic individuals due to its MASH-improving properties, although the long-term effects of treatment remain unknown [198,199].

Bariatric surgery emerges as a viable therapeutic choice for individuals meeting specific criteria for metabolic weight loss procedures, and it effectively resolves MASLD/MASH in most non-cirrhotic patients, reducing risk for CVD and malignancy [200]. A meta-analysis found that patients with obesity and CVD who underwent bariatric surgery had significantly lower odds of MACE compared to those who did not [201]. A recent study has observed the higher effectiveness of bariatric-metabolic surgery in the management of MASH as compared with lifestyle interventions and optimized medical care [202]. Patients with end-stage liver disease awaiting liver transplantation should undergo a thorough evaluation by a multidisciplinary team to identify and manage cardiovascular and metabolic comorbidities. This proactive approach minimizes the risk of major cardiovascular events throughout the pre-, peri-, and post-transplant phases [119].

### 5.3. Secondary Prevention of CVD

Secondary prevention in MASLD focuses on a comprehensive approach that includes managing risk factors for CVD, reducing long-term morbidity, and improving quality of life. Anti-obesity and antidiabetic medications, in addition to the lifestyle modifications discussed above, may offer potential benefits in both managing MASLD, its progression, and associated cardiometabolic factors. A recent study found that GLP-1 agonist use in patients with MASLD was associated with a reduced risk of major cardiovascular events, clinically significant portal HTN, and overall mortality [203]. Moreover, pharmacological treatments targeting dyslipidemia and HTN, two common comorbidities associated with MASLD and integral components of MetS, should be implemented according to established clinical guidelines.

Statins, as lipid-lowering drugs, are considered safe for patients with MASLD and those with compensated cirrhosis and should be prescribed to reduce the incidence of CVD when indicated [119,122]. A recent study showed that markers of MASLD severity in patients with familial hypercholesterolemia improved more in those receiving combination therapy with statins and the cholesterol absorption inhibitor ezetimibe, as well as in those treated with a regimen of statins, ezetimibe, and PCSK9 inhibitors, compared to those on statin monotherapy [204]. However, long-term data are required to better evaluate the sustained clinical impact of these treatments in MASLD patients. Bempedoic acid, an adenosine triphosphate-citrate lyase inhibitor, is a novel lipid-lowering therapy that has demonstrated promising effects in preclinical models of MASLD. Nevertheless, further clinical trials are crucial to thoroughly assessing its efficacy and safety in MASLD patients [205]. In MASLD patients with severe hypertriglyceridemia (>500 mg/dL), fibrates alone or with omega-3s or icosapent ethyl help reduce pancreatitis risk. They may also improve ASCVD outcomes when triglycerides are ≥200 mg/dL and HDL-C < 40 mg/dL [122].

HTN management involves a combination of lifestyle modifications, including a diet with reduced sodium intake, alongside the use of five primary classes of antihypertensive medications: angiotensin-converting enzyme inhibitors, angiotensin receptor blockers, beta-blockers, calcium channel blockers, and diuretics. This multifaceted approach has been demonstrated to not only effectively reduce BP but also decrease the risk of cardiovascular events in individuals without established ASCVD. The extent of benefit is closely linked to the degree of BP reduction achieved [206].

In summary, effectively managing MASLD requires aggressive treatment of its MetS components, timely diagnosis and management of comorbidities, and adherence to a healthy lifestyle. A comprehensive strategy, including lifestyle modifications, weight loss, medication optimization, and, when necessary, a combination of conservative and surgical therapies, is crucial for reducing both liver-related and cardiovascular-related risks. Table 4 summarizes the past, present, and future of MASLD management.

## 6. Conclusions

MetS and MASLD are two often-coexisting clinical conditions that share common underlying metabolic abnormalities. This clinical combination poses challenges in terms of treatment, as multiple expertise and a holistic approach are warranted for improving patients’ outcomes. Tailored strategies for the factors contributing to MetS and MASLD are crucial for reducing both cardiac and liver disease-related adverse events. Early promotion of lifestyle modifications, including adopting a healthy diet, engaging in regular physical activity, and managing body weight, is the first goal to achieve for improving patients’ outcomes. Moreover, early detection and pharmacologic treatment hold the potential of contrasting disease progression and reducing the risk of complications associated with MASLD and MetS.

Advancements in non-invasive diagnostics, the integration of social determinants of health, precision medicine approaches, and innovative pharmacological therapies are potential innovative avenues to pursue in the MASLD research agenda. Incretin-based agents and antifibrotic agents are expected to play a key role in transforming MASLD management. Additionally, research into the “gut-liver-muscle-pancreas-adipose tissue-central nervous system axis”, digital health innovations, and genetic factors may pave the way for more personalized therapies [119,122,207]. As MASLD has been suggested as a distinct non-communicable chronic disease [208], further research studies are warranted in the near future. Finally, large-scale holistic interventions addressing obesity and lifestyle modifications will be instrumental in mitigating disease burden and improving patient outcomes.

## Figures and Tables

**Table 1:** Definitions of NAFLD, MAFLD, MASLD.

Characteristics	Non-Alcoholic Fatty Liver Disease (NAFLD)	Metabolic Dysfunction-Associated Fatty Liver Disease (MAFLD)	Metabolic Dysfunction-Associated Steatotic Liver Disease (MASLD)
Hepatic steatosis > 5% assessed by liver histology or imaging techniques	✔	✔	✔
Hepatic steatosis > 5% assessed through blood biomarkers/scores	X	✔	X
Absence of significant alcohol consumption (≥30 g/day in men, ≥20 g/day in women)	✔	X	✔
Exclusion of other causes of steatosis	✔	X	✔
Presence of a cardiometabolic criteria (metabolic dysfunction)	X	✔	✔
		1. Overweight/Obesity (BMI ≥ 25 kg/m^2^ in Caucasians [23 kg/m^2^ in Asians]) 2. T2D (Based on widely recognized international guidelines) 3. Lean/normal weight (BMI < 25 kg/m^2^ in Caucasians or BMI < 23 kg/m^2^ in Asians) if present at least two metabolic abnormalities: A—WC ≥ 102 cm (M), ≥88 cm (F) in Caucasians (or ≥90 cm (M), ≥88 cm (F) in Asians B—Plasma HDL–C ≤ 40 mg/dL [1 mmol/L] (M) and ≤50 mg/dL [1.3 mmol/L] (F) OR lipid-lowering treatment C—Plasma TG ≥ 150 mg/dL [1.70 mmol/L] OR lipid-lowering treatment D—BP ≥ 130/85 mmHg OR specific antihypertensive drug treatment E—Prediabetes (Fasting Serum Glucose ≥ 100 to 125 mg/dL [5.6 to 6.9 mmol/L] OR 2 h post-load glucose levels ≥ 140 to 199 mg/dL [7.8 to 11.0 mmol/L] OR HbA1c ≥ 5.7 to 6.4% [39 to 47 mmol/mol]) F—Homeostasis model assessment of insulin resistance score ≥ 2.5 G—Plasma high sensitivity C-reactive protein level > 2 mg/L	BMI ≥ 25 kg/m^2^ [23 kg/m^2^ in Asians] OR WC > 94 cm (M) 80 cm (F) OR ethnicity adjusted measurements Fasting Serum Glucose ≥ 100 mg/dL [5.6 mmol/L] OR 2 h post-load glucose levels ≥ 140 mg/dL [7.8 mmol/L] OR HbA1c ≥ 5.7% [39 mmol/mol] OR T2D OR treatment for T2D Plasma HDL–cholesterol ≤ 40 mg/dL [1 mmol/L] (M) and ≤50 mg/dL [1.3 mmol/L] (F) OR lipid-lowering treatment Plasma TG ≥ 150 mg/dL [1.70 mmol/L] OR lipid-lowering treatment BP ≥ 130/85 mmHg OR specific antihypertensive drug treatment
Embraces the concept of a multifactorial aetiology	X	✔	✔

Abbreviations: BMI, body mass index; BP, blood pressure; WC, waist circumference; HDL-C, high-density lipoprotein cholesterol; TG, triglycerides; HbA1c, Hemoglobin A1c; M, males; F, females; T2D, type 2 diabetes.

**Table 2:** Definitions of metabolic syndrome.

	National Cholesterol Education Program/Adult Treatment Panel III	International Diabetes Federation	Unifying Metabolic Syndrome Statement
**Criteria**	At least 3 of the following criteria	Central obesity, plus at least 2 of the following criteria	At least 3 of the following criteria
*Waist Circumference/Obesity*			
	Abdominal obesity ^a^ *Men ≥ 102 cm (40 in)* *Women ≥ 88 cm (35 in)*	Central Obesity * *WC—ethnicity specific:* *a. Europids, Sub-Saharan Africans, Middle East, Eastern Mediterranean* *≥94 cm (M), ≥80 cm (F)* *b. South Asians, Chinese, Ethnic central and South Americans* *≥90 cm (M), ≥80 cm (F)* *c. Japanese* *≥85 cm (M), ≥90 cm (F)*	Elevated WC ** *Population specific and country specific definitions*
*Fasting plasma glucose*			
≥100 mg/dL (5.6 mmol/L)	✔ ^b^	✔	✔
*Or*			
prior T2D diagnosis	X	✔	X
*Or*			
pharmacological therapy for high glucose	X	X	✔
*High-density lipoprotein cholesterol*			
Men < 40 mg/dL (1.04 mmol/L) Women < 50 mg/dL (1.30 mmol/L)	✔	✔	✔
*Or*			
Targeted therapy for this lipid disorder	X	✔	✔
*Triglycerides*			
≥150 mg/dL (1.7 mmol/L)	✔	✔	✔
*Or*			
Targeted therapy for this lipid disorder	X	✔	✔
*Blood Pressure*			
SBP ≥ 130 mmHg	✔	✔	✔
*Or*			
DBP ≥ 85 mmHg	✔	✔	✔
*Or*			
Treatment for HTN	X	✔	✔

Table legend: Abbreviations: T2D, type 2 diabetes; HTN, hypertension; SBP, systolic blood pressure; DBP, diastolic blood pressure; WC, waist circumference. ^a^ Some male patients may develop multiple metabolic risk factors with even a modest increase in WC, such as from 94 to 102 cm (37 to 39 in). This suggests a possible strong genetic predisposition to insulin resistance. For these individuals, lifestyle changes can be as beneficial as they are for those with more substantial increases in WC. ^b^ In 2001, an elevated fasting plasma glucose level was defined as being ≥6.1 mmol/L (110 mg/dL). However, in 2004, this criterion was revised to ≥5.6 mmol/L (100 mg/dL), aligning it with the updated definition of IFG by the American Diabetes Association. * If the BMI is >30 kg/m^2^, central obesity can be assumed, eliminating the need for WC measurement. ** It is recommended to use IDF cut points for non-Europeans and either IDF or AHA/NHLBI cut points for individuals of European origin, until additional data are available.

**Table 3:** Non-invasive tests for assessing liver fibrosis in MASLD: pros and cons.

**Test**	**Advantages**	**Disadvantages**
Fibrosis index based on 4 factors (FIB-4) score FIB-4 score = age (years) × AST (U/L)/(platelet count (109/L) × √ALT (U/L)	-Easy to calculate -Commonly measured parameters -Widely validated -Cost-effective -Good alternative for initial screening	-Reduced accuracy in specific populations (individuals under 35 years of age, those over 65, people with significant alcohol consumption, and patients with other underlying liver conditions). -No information on aetiology -Not perfect for early-stage fibrosis -Accuracy influenced by coexisting conditions
NAFLD fibrosis score (NFS) NFS = −1.675 + 0.037 × age (years) + 0.094 × BMI (kg/m^2^) + 1.13 × impaired fasting glucose/diabetes (yes = 1, no = 0) + 0.99 × (AST/ALT ratio) − 0.013 × platelet count (×109/L) − 0.66 × albumin (g/dL)	-Easy to calculate -Commonly measured parameters -Widely validated -Cost-effective -Good alternative for initial screening	-Reduced accuracy in specific populations (individuals with very high or very low BMI, significant alcohol consumption, or other underlying liver diseases). -No information on aetiology -Not perfect for early-stage fibrosis -Accuracy can be influenced by coexisting conditions -Requires multiple variables
Enhanced liver fibrosis (ELF) score ELF score = 2.278 + 0.851 ln (hyaluronic acid) + 0.751 ln (PIIINP) + 0.394 ln (TIMP-1)	-Widely validated -High accuracy for detecting fibrosis	-Cost -No information on aetiology -Not perfect for early-stage fibrosis -Accuracy affected by coexisting conditions (obesity, diabetes, kidney disease) -Limited availability
Liver Stiffness Measurement-Vibration Controlled Transient Elastography Liver (LSM- VCTE) [FibroScan] It assesses liver stiffness using ultrasound-based elastography (e.g., FibroScan), with values measured in kilopascals (kPa), and liver steatosis using the Controlled Attenuation Parameter (CAP), with values measured in decibels per meter (dB/m).	-Widely validated -High accuracy for detecting fibrosis -Quick and easy with real-time results	-Cost -No information on aetiology -Not perfect for early-stage fibrosis -Accuracy can be impacted by coexisting conditions, such as obesity, ascites, liver congestion, and others. -Possible operator dependence -Limited availability
FibroScan-AST (FAST) score FAST = VCTE [Liver Stiffness Measurement (LSM), Controlled Attenuation Parameter (CAP)] + AST FAST score = e^−1.65 + 1.07 × ln (LSM) + 2.66*10^−8^ × CAP^3^ − 63.3 × AST^−1^^/1 + e^−1.65 + 1.07 × ln (LSM) + 2.66*10^−8^ × CAP^3^ − 63.3 × AST^−1^^	-Widely validated -Enhanced accuracy for detecting fibrosis -Quick and easy	-Cost -No information on aetiology -Not perfect for early-stage fibrosis -Accuracy can be impacted by coexisting conditions, such as obesity, ascites, liver congestion, and others. -Possible operator dependence -Limited availability
Magnetic resonance imaging (MRI), MRI proton density fat fraction (MRI-PDFF) measures liver steatosis Magnetic resonance elastography (MRE) (Standard MRI machines utilizing a phase contrast technique, along with specialized software, to evaluate liver stiffness by analysing the propagation of mechanical waves through the liver tissue. MRI-iron-corrected T1 mapping (cT1)	-Widely validated -Highly accurate for detecting fibrosis -Better for heterogeneous liver disease -Provides detailed imaging of the entire liver -Can be combined with other parameters (MAST = MRE + MRI-PDFF + AST; MEFIB = MRE + FIB-4)	-Cost -No information on aetiology -Requires specialized equipment and expertise -Limited availability

Abbreviations: AST, aspartate aminotransferase; ALT, alanine aminotransferase; HA, hyaluronic acid; PIIINP, procollagen III amino-terminal peptide; TIMP-1, tissue inhibitor of matrix metalloproteinase type 1.

**Table 4:** The past, the present and the future of MASLD management.

Area	Past	Present	Future
Treatment	Lifestyle Modifications Management of MetS components, comorbidities, and cardiovascular disease.	Lifestyle Modifications Stronger emphasis on managing MetS components with preferred pharmacologic treatments, addressing comorbidities, and implementing an integrated approach to cardiovascular risk.	Lifestyle Modifications Stronger emphasis on managing MetS components with personalized pharmacologic treatments, addressing comorbidities, and implementing an integrated approach to cardiovascular risk.
	No specific drug approved	Resmetirom (Only in US)	Resmetiron (global use) Potential approval of new drugs, including Semaglutide, Tirzepatide, Survodutide, Retatrutide, Efruxifermin, Pegozafermin, and Lanifibranor. Combination therapy Specific treatments for individuals with MASH- related cirrhosis
	Bariatric surgery Liver transplantation	Bariatric surgery Liver transplantation	Bariatric surgery Liver transplantation

## Data Availability

Data sharing is not applicable to this article as no datasets were generated or analyzed during the current study.
